# Correlation analysis and prediction models for loess compressibility in Ili region, Xinjiang

**DOI:** 10.1371/journal.pone.0345028

**Published:** 2026-03-23

**Authors:** Zhiqi Liu, Lifeng Chen, Kai Chen, Zizhao Zhang, Jinyu Chang

**Affiliations:** 1 School of Geology and Mining Engineering Xinjiang University, Urumqi, Xinjiang, China; 2 Key Laboratory of Green and Efficient Mining and Ecological Restoration in High-Altitude Arid Regions of Xinjiang, Urumqi, Xinjiang, China; 3 School of Resource and Earth Science, China University of Mining & Technology, Xuzhou, Jiangsu, China; 4 Xinjiang Institute of Ecology and Geography, Chinese Academy of Sciences, Urumqi, Xinjiang, China; China University of Mining and Technology, CHINA

## Abstract

Loess compressibility is a crucial engineering parameter governing the deformation of loess foundations and the evolution of slope geohazards. Based on a comprehensive collection of physical, hydraulic, and mechanical parameters of loess in the Ili region, this study selected Huocheng, Nilka, and Xinyuan counties as typical study areas. Statistical methods were employed to a perform normality tests and necessary transformations on the data, followed by correlation analysis to identify key factors influencing the compression coefficient. Using Multiple Linear Regression (MLR) as a baseline, six machine learning models were constructed, including Random Forest (RF), Multilayer Perceptron (MLP), Radial Basis Function (RBF), Support Vector Machine (SVM), Classification and Regression Tree (CART), and XGBoost models. The results indicate that the compression coefficient is significantly positively correlated with the void ratio and negatively correlated with dry density and compressibility modulus. Consequently, compressibility modulus, dry density, and void ratio were selected as core input indicators. All constructed models successfully predicted the compression coefficient and its engineering classification. Under the evaluation principle of “error metrics priority, classification accuracy auxiliary,” the MLP model achieved the best overall performance across the three counties, followed by the Random Forest model. This study provides a methodological basis for the rapid estimation of loess compressibility parameters and engineering judgment in the Ili region.

## 1. Introduction

Loess compressibility refers to its ability to undergo volumetric compression deformation under external loads, influenced by factors such as mineral composition, particle size distribution, pore structure, and water content. Loess is widely distributed in the Ili region. Under inducing factors such as self-weight, rainfall, and human engineering activities, it is prone to compression deformation, directly impacting the bearing capacity and settlement characteristics of foundations. This holds significant importance for the stability and safety of infrastructure like buildings, roads, and bridges. Therefore, scientifically selecting parameters to construct loess compressibility prediction models is crucial, as it provides an important scientific basis for practical engineering construction and geological hazard prevention and control.

The correlation analysis between loess compressibility and soil properties forms the foundation for establishing its prediction models. Researchers domestically and internationally have explored the compression characteristics of loess from different regions, types, and states, along with their influencing factors, using various methods and experiments. They have developed mathematical models or empirical formulas based on statistical and mechanistic analyses to quantify the relationship between loess compressibility indicators and its physical and mechanical properties. Jiang et al. [1] utilized the Discrete Element Method (DEM) to simulate one-dimensional compression and wetting tests of loess, investigating the influence of water content and void ratio on loess compression and collapse. Mu et al. [2] employed SEM, XRD, and MIP tests, alongside triaxial compression tests, to study the correlation between the compression properties of undisturbed and compacted loess and their structure and pore size. Chen et al. [3] constructed multiple linear regression and RBF neural network models for loess collapsibility prediction in Gongliu County, Ili River Valley, based on correlation analysis. Yuan et al. [4] quantitatively explored the correlation between the modulus of compressibility and collapsibility of loess in southern Shanxi Province through indoor compression tests. Jian [5] fitted e-lgp curves for Malan loess with different dry densities, water contents, and regions using Gregory’s logarithmic function model and analyzed the correlation.

Prediction models for loess compressibility are vital tools for assessing and managing loess settlement issues. Prediction models based on machine learning methods offer significant advantages in handling vague, stochastic, and nonlinear data [6,7]. Chen et al. [8] developed prediction models for loess collapsibility in Xinyuan County, Ili region, using multiple linear regression and neural network theories. Xu [9] constructed a 1D loess compression model based on the Disturbed State Concept (DSC) theory, defining the disturbance function using the void ratio as a parameter. C. A. A. et al. [10] proposed a nonlinear regression model to predict parameters such as the secondary compression index, liquid limit, plastic limit, and water content for evaluating soil compression characteristics. Shi et al. [11] conducted high-pressure consolidation tests on remolded loess in different states with varying water contents and dry densities, establishing settlement prediction models for filled sites of different thicknesses (loads). Huang et al. [12] developed multifactor regression models for loess collapsibility coefficient using ordinary multiple linear regression and partial least squares regression methods. Ma et al. [13] analyzed correlations using loess from central Shanxi Province as an example, then quantitatively ranked them by partial correlation analysis, and constructed an RBF neural network model. Zhang [14] compared the accuracy of RBF neural network models and Newrbe functions through modeling factor analysis, verified by bivariate two-tailed correlation analysis. Zhan [15] and Gao et al. [16] established BP neural network prediction models for loess collapsibility coefficient using samples from the Dingxi-Lintao Expressway project and Xi’an loess, respectively, based on geotechnical test parameters.

Formed by the complex interaction of glacial transport and aeolian deposition, Ili loess exhibits distinct genesis, collapsibility, and soluble salt content compared to the typical loess of other regions [17–19]. Despite the maturity of research on the Loess Plateau, research specifically targeting the compressibility mechanisms of Ili loess remains sparse. Furthermore, the comparative applicability of advanced machine learning algorithms in this specific geological context has not been fully explored. Given the significant spatial variability and non-linear physical-mechanical characteristics of Ili loess, traditional prediction models based on simple physical relationships or linear regression often fail to accurately capture the multi-factor coupling effects. Therefore, focusing on the loess in the Ili region, this study utilizes a comprehensive dataset of geotechnical parameters. We first employ multivariate statistical theory to analyze the correlation between soil properties and the compression coefficient, optimizing the input parameters for prediction models. Subsequently, multiple regression and various machine learning models are constructed and compared to identify the optimal prediction tool, thereby providing a robust scientific basis for engineering construction and geohazard prevention in the Ili region.

## 2. Study area overview and data source

### 2.1. Study area and sampling strategy

The Ili region is located in western Xinjiang, China. The terrain generally slopes from east to west, with high elevations in the north and south, and low elevations in the center. East-west trending mountainous belts, with altitudes between 1000 - 5000m, are distributed on both northern and southern sides and in the central part. The central area comprises the Ili River, Kashi River, Gongnaisi River valleys, and the Tekes River valley, with altitudes between 500 - 1000m. The Ili region has a diverse climate, with an average annual temperature ranging from 2.6 - 10.4 °C. Precipitation in the Ili region is relatively abundant but unevenly distributed due to topography; the average annual precipitation in plain areas is 200–500 mm, while mountainous areas can exceed 800 mm, with a spatial trend of higher rainfall in the east than in the west [19]. As a western sub-region of loess distribution in Xinjiang, loess in the Ili region is widely distributed. Situated in the westernmost loess belt of Xinjiang, the Ili region exhibits a distinctive depositional pattern governed by the topographic interception of the Westerlies. The spatial distribution is characterized by lenticular accumulation along the river valleys, presenting a typical ‘thick-center, thin-margin’ profile. In terms of granulometry, a clear aeolian sorting gradient is observed, with grain size fining from west to east, reflecting the diminishing transport energy of the prevailing winds [20,21].

**Fig 1:**
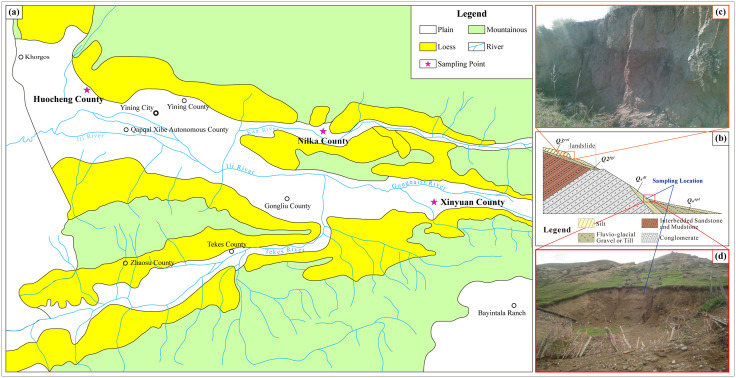
Overview of study area and schematic diagram of sampling locations. **(a)**General distribution map of loess in Ili region (Schematic redrawn based on geological information from Ye et al. [20]); **(b)**Typical geological cross-section of loess strata; **(c)**Field photograph of Upper Pleistocene aeolian loess on slope surface; **(d)**Field photograph of Holocene colluvial loess at slope toe.

A preliminary statistical analysis of geotechnical parameters collected from various geological hazard projects across the Ili region revealed significant data variability and indistinct correlations. To mitigate this noise and capture representative compressibility characteristics, this study adopted a sampling strategy guided by the spatial distribution of loess in the Ili River Valley. Specifically, representative samples were selected from Huocheng County (west), Nilka County (central), and Xinyuan County (east) to highlight typical geotechnical relationships. The general distribution of loess and specific sampling locations are illustrated in Fig 1(a). Additionally, Fig 1(b) presents a typical geological cross-section of the strata, while Fig 1(c) and 1(d) display representative field photographs of the sampling sites.

Probabilistic statistical analysis of the physical and mechanical parameters of loess in the study area was conducted to obtain characteristic statistical quantities such as mean, standard deviation, and skewness, which characterize the spatial randomness of geotechnical parameters. The results of the probabilistic statistical analysis for loess strata parameters in the study area are presented in Table 1.

**Table 1:** Probabilistic statistical analysis results of loess geotechnical parameters.

Soil Property (Unit^a^)		Huocheng County			Nilka County			Xinyuan County		
		Mean	Std. Dev.	Skewness	Mean	Std. Dev.	Skewness	Mean	Std. Dev.	Skewness
**Density (g/cm**^**3**^)	*ρ*	1.560	0.131	0.940	1.783	0.172	−0.435	1.558	0.208	0.208
**Dry Density (g/cm**^**3**^)	*ρ*_*d*_	1.437	0.080	−0.606	1.483	0.121	0.301	1.379	0.156	0.156
**Void Ratio (–)**	*e*	0.886	0.110	0.887	0.833	0.152	0.343	0.977	0.211	0.211
**Porosity (%)**	*n*	46.800	2.975	0.641	45.065	4.526	−0.302	48.810	5.807	5.807
**Water Content (%)**	*ω*	8.495	6.048	1.023	20.203	5.961	−0.324	7.213	8.001	3.229
**Liquid Limit (%)**	*ω*_*L*_	26.150	0.707	2.340	26.591	1.775	−1.593	26.691	1.409	1.409
**Liquidity Index (–)**	*I*_*L*_	−0.982	0.692	1.056	0.275	0.596	−0.228	−0.534	0.563	0.563
**Plastic Limit (%)**	*ω*_*P*_	17.259	0.467	2.340	17.550	1.172	−1.595	17.687	1.276	1.276
**Plasticity Index (%)**	*I*_*P*_	8.891	0.241	2.328	9.040	0.603	−1.597	9.008	0.653	0.653
**Modulus of Compressibility (MPa)**	*E*_*s*_	14.729	7.308	0.647	7.986	4.322	0.993	10.233	6.416	6.416
**Collapsibility Coefficient (–)**	*δ*_*s*_	0.030	0.032	1.315	0.020	0.033	2.514	0.066	0.048	0.048
**Self-weight Collapsibility (–)**	*δ*_*zs*_	0.016	0.028	5.045	0.007	0.012	2.599	0.040	0.055	0.055
**Natural Internal Friction Angle (°)**	*φ*	26.446	6.692	−2.623	23.973	7.838	−1.488	23.322	6.175	6.175
**Saturated Internal Friction Angle (°)**	*φ’*	25.948	3.462	0.645	24.599	4.399	−1.317	23.848	4.940	4.940

^a^ “–”indicates dimensionless parameters.

### 2.2. Research methods

#### 2.2.1. Statistical analysis and parameter optimization.

Based on the compiled data, IBM SPSS Statistics software was used to perform descriptive statistics and normality tests (Kolmogorov-Smirnov test combined with Q-Q plots) on the physical-mechanical parameters of loess within the Ili region. For data that did not satisfy normal distribution, logarithmic transformation was prioritized for positive variables, while Johnson system transformation [22] was applied to other skewed data for preprocessing, thereby reducing the influence of data skewness on parameter statistics and correlation analysis [23–25]. On this basis, Pearson correlation analysis [25] was employed to quantitatively evaluate the linear correlation degree between various soil properties and the compression coefficient. Based on the correlation coefficients and significance levels, the most representative key influencing factors were screened out to serve as input variables (feature vectors) for the prediction models. In this study, a Pearson correlation coefficient (*R*) with an absolute value between 0.8-1.0 is considered extremely strong correlation, 0.6-0.8 as strong correlation, 0.4-0.6 as moderate correlation, 0.2-0.4 as weak correlation, and 0.0-0.2 as very weak or no correlation, with 0.05 set as the significance level for probability standards.

#### 2.2.2. Prediction model construction and evaluation.

Given that loess compression deformation is controlled by the non-linear coupling of multiple physical state parameters, this study constructed a model evaluation system using Multiple Linear Regression (MLR) neural network as the baseline, Random Forest (RF) and Multilayer Perceptron (MLP) as the core, and Radial Basis Function (RBF) neural network, Support Vector Machine (SVM), Classification and Regression Tree (CART), and eXtreme Gradient Boosting (XGBoost) for comparison.

First, the MLR model was constructed as the evaluation benchmark. While MLR has the advantage of clear physical meaning, its nature based on linear assumptions makes it difficult to accurately capture the complex non-linear features among high-dimensional geotechnical parameters; therefore, this study mainly used it as a reference baseline to measure the performance improvement of machine learning models. Addressing the non-linear regression needs of loess parameters, this study focused on introducing and tuning two core algorithms: RF and MLP. As a representative of ensemble learning, RF constructs multiple regression trees via Bootstrap resampling [26–29]; meanwhile, it introduces random feature subsets in node splitting to reduce inter-tree correlation, thereby reducing the variance and overfitting risk of single decision trees and enhancing robustness to noise and outliers; during modeling, hyperparameters such as the number of trees and maximum depth were optimized via grid search to obtain better generalization performance [30,31]. As a typical feedforward artificial neural network model, MLP consists of an input layer, several hidden layers, and an output layer. It achieves non-linear mapping from input soil indices to the predicted compression coefficient through non-linear activation. Under the backpropagation framework, optimization strategies such as quasi-Newton or conjugate gradient methods are adopted to iteratively update network weights and bias parameters [32–35] to characterize the complex non-linear relationship between the compression coefficient and multiple indices. In addition, to ensure the robustness of the model selection conclusion, this study also introduced four mainstream algorithms with different mechanisms including RBF, SVM, CART, and XGBoost, for cross-validation comparison.

In the model training and evaluation stage, based on the IBM SPSS Modeler platform, the three regions were modeled and evaluated independently. By comparing the result errors of each model on the test set and combining them with the accuracy of engineering classification, the model with the best comprehensive performance was finally selected under the sample and indicator system conditions of this study.

## 3. Loess prediction model establishment

### 3.1. Selection of soil properties

Based on the collected geotechnical parameters, normality tests and necessary data transformations (logarithmic or Johnson system transformation) were performed following the methods described in Section 2.2 to satisfy the prerequisites for linear correlation analysis. The results of the correlation analysis are presented in Table 2.

**Table 2:** Statistical results of correlation analysis between loess compression coefficient and soil properties.

Classification	Soil Property		Huocheng County			Nilka County			Xinyuan County		
			Corr. Coeff.	Correlation	*Sig.*	Corr. Coeff.	Correlation	*Sig.*	Corr. Coeff.	Correlation	*Sig.*
**Physical Prop.**	Density	*ρ*	−0.181	Weak	0.229	−0.579	Moderate	<0.001^	−0.45^b^	Moderate	<0.001^
	Dry Density	*ρ*_*d*_	−0.625	Strong	<0.001^	−0.665	Strong	<0.001^	−0.662^b^	Strong	<0.001^
	Void Ratio	*e*	0.666	Strong	<0.001^	0.722	Strong	<0.001^	0.678^b^	Strong	<0.001^
	Porosity	*n*	0.625	Strong	<0.001^	0.661	Strong	<0.001^	0.669^b^	Strong	<0.001^
	Water Content	*ω*	0.433^a^	Moderate	0.003^	−0.048	Very Weak	0.682	0.258^b^	Strong	<0.001^
	Saturation	*S*_*r*_	0.322^a^	Weak	0.029	−0.335	Weak	0.003^	0.042^b^	Very Weak	0.552
**Hydraulic Prop.**	Liquid Limit	*ω*_*P*_	0.260	Weak	0.081	0.068	Very Weak	0.561	0.238	Weak	<0.001^
	Plasticity Index	*I*_*L*_	0.360	Weak	0.014^	−0.053^a^	Very Weak	0.649	0.232	Weak	<0.001^
	Plastic Limit	*ω*_*L*_	0.260	Weak	0.081	−0.068	Very Weak	<0.001^	0.215^b^	Weak	0.002^
	Plasticity Index	*I*_*P*_	0.262	Weak	0.079	−0.068	Very Weak	0.562	0.067^b^	Very Weak	0.348
**Mechanical Prop.**	Modulus of Compressibility	*E*_*s*_	−0.902^a^	Extremely Strong	<0.001^	−0.892^a^	Extremely Strong	<0.001^	−0.912^b^	Extremely Strong	<0.001^
	Collapsibility Coefficient	*δ*_*s*_	0.349^a^	Weak	0.017	0.530	Moderate	<0.001^	0.133	Very Weak	0.060
	Self-weight Collapsibility	*δ*_*zs*_	0.211^a^	Weak	0.159	0.153	Very Weak	0.189	−0.147	Very Weak	<0.001^
	Natural Internal Friction Angle	*φ*	0.221^a^	Weak	0.149	0.002	Very Weak	0.987	−0.105	Very Weak	0.138
	Saturated Internal Friction Angle	*φ’*	0.248	Weak	0.097	−0.064	Very Weak	0.587	−0.085^b^	Very Weak	0.231

^a^ Indicates common logarithm (base 10) transformed data;

^b^ Indicates Johnson transformed data;

^Indicates that the correlation is significant at the 0.05 level (2-tailed).

**Fig 2:**
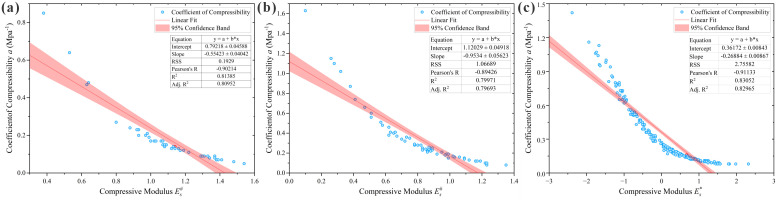
Scatter plots of loess compression coefficient vs. compression modulus correlation analysis. **(a)** Huocheng County; **(b)** Nilka County; **(c)** Xinyuan County.

**Fig 3:**
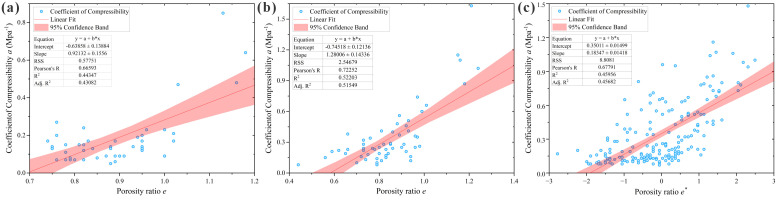
Scatter plots of loess compression coefficient vs. void ratio correlation analysis. **(a)** Huocheng County; **(b)** Nilka County; **(c)** Xinyuan County.

**Fig 4:**
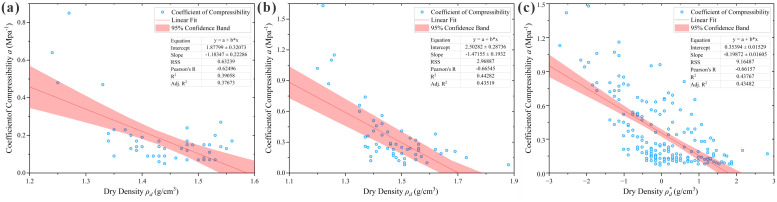
Scatter plots of loess compression coefficient vs. dry density correlation analysis. **(a)** Huocheng County; **(b)** Nilka County; **(c)** Xinyuan County.

As indicated in Table 2, significant linear correlations exist between the compression coefficient and dry density, porosity, void ratio, and modulus of compressibility across all three regions, verifying the reliability of the analysis results. Among these, the modulus of compressibility exhibits an extremely strong correlation, while void ratio, dry density, and porosity show strong correlations. Since void ratio and porosity are interconvertible indicators both reflecting pore properties, the modulus of compressibility (*E*_*s*_), dry density (*ρ*_*d*_), and void ratio (*e*) were ultimately selected as the three optimal input parameters for the prediction models. The scatter plots of their correlation analysis are presented in Figs 2–4, and the selected correlation coefficients are summarized in Table 3.

**Table 3:** Statistical table of selected soil property correlation coefficients.

Geotechnical Parameter	Pearson Correlation Coefficient with Compression Coefficient					
	Huocheng County		Nilka County		Xinyuan County	
**Dry density *ρ***_***d***_	−0.625	Strong correlation	−0.665	Strong correlation	−0.662	Strong correlation
**Void ratio *e***	0.666	Strong correlation	0.723	Strong correlation	0.678	Strong correlation
**Modulus of** **Compressibility *E***_***s***_	−0.902	Extremely strong correlation	−0.892	Extremely strong correlation	−0.912	Extremely strong correlation

### 3.2. Multiple regression prediction model

Multiple regression prediction models were constructed for each region, with a total of 320 sets of selected geotechnical parameter data as input. The independent variables were dry density (*ρ*_*d*_), void ratio (*e*), and the logarithm of modulus of compressibility (*E*_*s*_), while the dependent variable was the compression coefficient (*a*). Summaries of the established multiple regression prediction models are presented in Table 4 and Table 5.

**Table 4:** Summary of loess compressibility and soil property regression models.

Model	R	R^2^	Adjusted R^2^	Standard Error of the Estimate	Durbin-Watson
**Huocheng County**	0.942	0.887	0.879	0.05285	1.776
**Nilka County**	0.951	0.905	0.901	0.08438	1.899
**Xinyuan County**	0.932	0.869	0.867	0.104516	1.851

**Table 5:** Regression coefficients and significance analysis for loess compressibility and soil properties.

Model		Unstandardized Coefficients		Standardized Coefficients	t	
		*B*	Std. Error	*Beta*		Sig.
**Huocheng County**	(Constant)	−7.647	2.611		−2.929	0.005
	*ρ*_*d*_	3.797	1.234	2.005	3.078	0.004
	*E*	3.190	0.924	2.306	3.451	0.001
	*Es* ^*#*^	−0.412	0.042	−0.670	−9.785	<0.001
**Nilka County**	(Constant)	−8.793	1.370		−6.419	<0.001
	*ρ*_*d*_	4.294	0.627	1.942	6.846	<0.001
	*E*	3.985	0.518	2.249	7.698	<0.001
	*Es* ^*#*^	−0.683	0.051	−0.641	−13.340	<0.001
**Xinyuan County**	(Constant)	0.350	0.008		44.618	<0.001
	*ρ*_d_^*^	0.474	0.131	1.578	3.610	<0.001
	*e* ^*^	0.489	0.120	1.806	4.083	<0.001
	*Es* ^*^	−0.223	0.010	−0.756	−22.804	<0.001

Tables 4 and 5 indicate that the independent variables in the three established models explain 87.9%, 90.1%, and 86.7% of the variation in the dependent variable, respectively, demonstrating excellent explanatory power. The data independence supports the assumption of residual independence, suggesting a very high goodness-of-fit for these predictive regression models. Furthermore, the significance (*p*-value) for all parameters (dry density *ρ_d_*, void ratio *e*, modulus of compressibility *E_s_*, and constant) in the established loess compressibility regression prediction models is less than 0.05, confirming that the selected parameters for these prediction models are statistically significant and reasonable.

Based on the pre-processing of loess parameters (dry density *ρ_d_*, void ratio *e*, and modulus of compressibility *E_s_*) in the study area, and combined with the statistical test results of the regression models, regression prediction models for loess compressibility were established for Huocheng County equation (1), Nilka County equation (2), and Xinyuan County equation (3), respectively.


$$a=-7.647+3.797\rho_{d}+3.190e-0.412lgE_{s}$$ (1)

$$a=-8.793+4.294\rho_{d}+3.985e-0.683lgE_{s}$$ (2)

$$a=0.350+0.474\rho_{d}^{*}+0.489e^{*}-0.223E_{s}^{*}$$ (3)

In equation (3), *ρ*_d_^*^, e^*^, and *E*_s_^*^ represent data processed by Johnson transformation. The transformation process is detailed in equations (4)–(6).


$$\rho_{d}^{*}=-0.816+2.053\times Asinh\left(\frac{\rho_{d}-1.256}{0.166}\right)$$ (4)

$$e^{*}=0.498+2.217\times Asinh\left(\frac{e-1.095}{0.258}\right)$$ (5)

$$E_{s}^{*}=0.801+0.666\times ln\left(\frac{E_{s}-1.407}{28.449-E_{s}}\right)$$ (6)

It is noteworthy that the models for Huocheng and Nilka adopt the same functional form with similar coefficient magnitudes, indicating a consistent response of compressibility to pore state and stiffness indices in these regions. In contrast, the Xinyuan model differs formally because the data required normalization via Johnson system transformation prior to linear regression, essentially representing a composite mapping of monotonic transformation and linear regression. Furthermore, since *ρ*_*d*_ and *e* both characterize pore geometric states and are statistically correlated, their regression coefficients in the multivariate model may differ in direction from univariate correlations due to multicollinearity, exhibiting higher sensitivity to the sample range.

**Fig 5:**
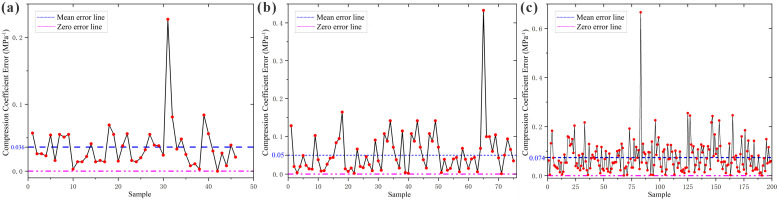
Error Plots of Loess Compressibility Multiple Regression Model Predictions vs. Measured Values. **(a)** Huocheng County; **(b)** Nilka County; **(c)** Xinyuan County.

The loess compressibility for each region was graded according to the “Code for Design of Building Foundation” (GB 50007−2011) [36] and compared with the predicted values from the established multiple regression models, as shown in Table 6 and Fig 5.

**Table 6:** Comparison of loess compressibility multiple regression model prediction results with measured data.

County	Prediction Status	Sample Size	Percentage (%)
**Huocheng County**	Accurate	44	95.65%
	Inaccurate	2	4.35%
**Nilka County**	Accurate	63	84.00%
	Inaccurate	12	16.00%
**Xinyuan County**	Accurate	170	85.43%
	Inaccurate	29	14.57%

As observed from Table 6, for the three regions, the number of samples where the multiple regression model’s predicted compressibility grade matched the actual compressibility grade was 44 for Huocheng County, achieving a prediction effectiveness of 95.65%; 63 for Nilka County, with an effectiveness of 84%; and 170 for Xinyuan County, with an effectiveness of 85.43%. Thus, the established multiple regression models are capable of predicting loess compressibility in the Ili region.

### 3.3. Random forest prediction model

Random Forest prediction models were constructed for each region, taking the selected geotechnical parameter dataset of 320 groups as input. The distribution and proportion of datasets for each sub-region are shown in Table 7.

**Table 7:** Dataset partitioning for random forest prediction model.

Partition	Huocheng County		Nilka County		Xinyuan County	
	No. of Samples	Proportion (%)	No. of Samples	Proportion (%)	No. of Samples	Proportion (%)
**Training Set**	34	73.9	57	76	133	66.8
**Test Set**	4	8.7	6	8	32	16.1
**Validation Set**	8	17.4	12	16	34	17.1
**Total**	46	100	75	100	199	100

**Fig 6:**
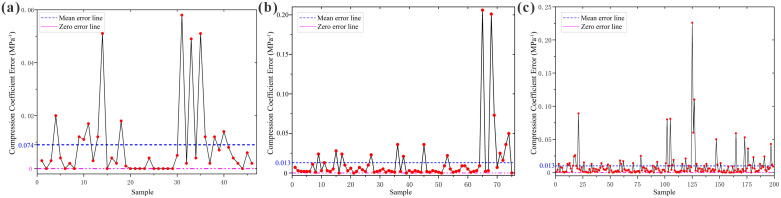
Error Plots of Loess Compressibility Random Forest Model Predictions vs. Measured Values. **(a)** Huocheng County; **(b)** Nilka County; **(c)** Xinyuan County.

The loess compressibility for each region was graded and compared with the predicted values from the established RF models, as shown in Table 8 and Fig 6.

**Table 8:** Comparison of loess compressibility random forest model prediction results with measured data.

County	Prediction Status	Sample Size	Percentage (%)
**Huocheng County**	Accurate	46	100
	Inaccurate	0	0
**Nilka County**	Accurate	74	98.67
	Inaccurate	1	1.33
**Xinyuan County**	Accurate	195	97.99
	Inaccurate	4	2.01

As observed from Table 8, for the three regions, the Random Forest model predicted compressibility grades identical to the actual compressibility grades for 46 samples in Huocheng County, achieving a prediction effectiveness of 100%; for 74 samples in Nilka County, with an effectiveness of 98.67%; and for 195 samples in Xinyuan County, with an effectiveness of 97.99%. Therefore, the established Random Forest models are capable of effectively predicting loess compressibility in the Ili region.

### 3.4. Neural network prediction model

MLP neural network prediction models were constructed for each region. The distribution and proportion of datasets for each sub-region are shown in Table 9.

**Table 9:** Dataset partitioning for multilayer perceptron neural network prediction model.

Partition	Huocheng County		Nilka County		Xinyuan County	
	No. of Samples	Proportion (%)	No. of Samples	Proportion (%)	No. of Samples	Proportion (%)
**Training Set**	26	56.5	57	76	147	73.9
**Test Set**	5	10.9	6	8	18	9
**Validation Set**	12	32.6	12	16	34	17.1
**Total**	46	100	75	100	199	100

**Fig 7:**
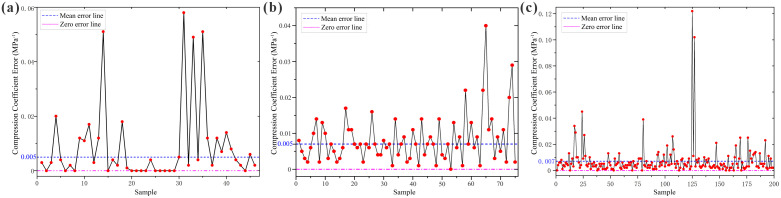
Error Plots of Loess Compressibility Multilayer Perceptron Neural Network Model Predictions vs. Measured Values. **(a)** Huocheng County; **(b)** Nilka County; **(c)** Xinyuan County.

The loess compressibility for each region was graded and compared with the predicted values from the established MLP neural network models, as shown in Table 10 and Fig 7.

**Table 10:** Comparison of loess compressibility multilayer perceptron neural network model prediction results with measured data.

County	Prediction Status	Sample Size	Percentage (%)
**Huocheng County**	Accurate	46	100
	Inaccurate	0	0
**Nilka County**	Accurate	75	100
	Inaccurate	0	0
**Xinyuan County**	Accurate	194	97.49
	Inaccurate	5	2.51

As observed from Table 10, for the three regions, the Multilayer Perceptron neural network model predicted compressibility grades identical to the actual compressibility grades for 46 samples in Huocheng County, achieving a prediction effectiveness of 100%; for 75 samples in Nilka County, with an effectiveness of 100%; and for 194 samples in Xinyuan County, with an effectiveness of 97.49%. Therefore, the established Multilayer Perceptron neural network models are capable of effectively predicting loess compressibility in the Ili region.

## 4. Discussion

### 4.1. Evaluation of indicator parameters

Loess compressibility is essentially a change in the soil pore structure, which is closely related to collapsibility and has significant implications for engineering construction [37]. Through the correlation analysis of soil properties, it was found that in all three selected regions, significant correlations exist between the compression coefficient (*a*) and dry density, porosity, void ratio, and modulus of compressibility. These parameters serve as effective indicators for establishing prediction models to more accurately assess loess compression properties under various conditions.

**Fig 8:**
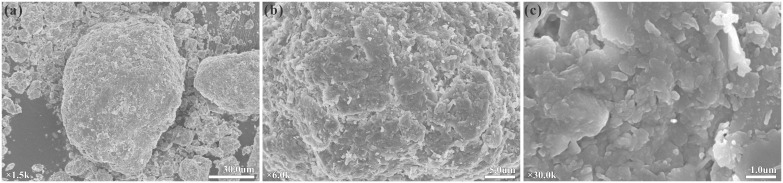
Scanning Electron Microscopy (SEM) images of Ili loess microstructure. **(a)** ×1500; **(b)** ×6000; **(c)** ×30000.

The strong correlation among void ratio, dry density, and compressibility align with the developed structural pore system and cementation-skeletal characteristics of Ili loess, as shown in the Scanning Electron Microscopy (SEM) images in Fig 8.

Combining the microstructural features in Fig 8, the loess in the study area typically develops a macroporous skeletal framework, where silt particles are loosely packed and strength is maintained primarily by soluble salts and clay mineral cementation. A higher void ratio implies a skeletal structure with abundant unstable point contacts, which are highly susceptible to yield and structural collapse under external loads, leading to significant macroscopic volume shrinkage. Conversely, a higher dry density indicates denser particle packing with enhanced interlocking effects, effectively restricting particle slippage and rearrangement under stress, thereby manifesting as lower compressibility.

The compression coefficient is a direct measure of loess compressibility and is closely related to water content and pore structure. The modulus of compressibility characterizes the stiffness response and strain change under confined conditions and is extremely strongly correlated with *a* (|R| > 0.9). As a stiffness parameter describing the stress-strain relationship, *E*_*s*_ integrates the macroscopic response of mineral composition, cementation strength, and stress history. In Ili loess, the combined action of carbonate and clay mineral cementation forms a structural skeleton, significantly enhancing the initial modulus. When loading exceeds the structural yield level, cementation bonds progressively break, potentially causing stiffness degradation.

In summary, the three selected indicators are not isolated statistical variables but characterize the key dimensions of compression response from “macroscopic stiffness,” “skeletal density,” and “pore structure potential,” providing a basis for the physical interpretability of the model inputs, which may also be one of the reasons for the high prediction accuracy of the MLP model.

### 4.2. Model comparison and analysis

**Fig 9:**
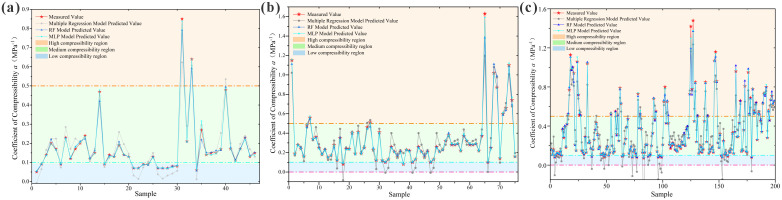
Comparison of Measured Compression Coefficient Values with Predicted Values from the Established Model. **(a)** Huocheng County; **(b)** Nilka County; **(c)** Xinyuan County.

The established loess compressibility prediction models were compared with measured results, as shown in Fig 9.

To select the optimal prediction model for loess compressibility in the Ili region, in addition to the previously mentioned regression model, Random Forest model, and MLP, RBF, SVM, CART, and XGBoost models were also established, totaling seven prediction models. The comparative results of the established models are presented in Table 11.

**Table 11:** Comparative statistical table of established prediction models for each region.

County	Model	Classification Accuracy	Mean Error (MPa ⁻ ¹)	Relative Error	Model Performance Rank
**Huocheng** **County**	MLP	100.00%	0.005	2.82%	1
	RF	100.00%	0.009	5.07%	2
	CART	100.00%	0.011	6.20%	3
	XGBoost	89.13%	0.015	8.46%	4
	Multiple Regression	95.65%	0.036	20.29%	5
	RBF	84.78%	0.036	20.29%	6
	SVM	93.48%	0.049	27.62%	7
**Nilka** **County**	MLP	100.00%	0.008	2.49%	1
	RF	100.00%	0.013	4.05%	2
	XGBoost	96.00%	0.016	4.98%	3
	CART	94.67%	0.019	5.92%	4
	Multiple Regression	95.65%	0.036	11.21%	5
	RBF	84.78%	0.047	14.63%	6
	SVM	94.67%	0.053	16.50%	7
**Xinyuan** **County**	MLP	97.49%	0.007	1.98%	1
	RF	98.99%	0.01	2.82%	2
	XGBoost	98.49%	0.018	5.08%	3
	CART	92.46%	0.02	5.65%	4
	Multiple Regression	84.42%	0.056	15.81%	5
	RBF	91.96%	0.069	19.48%	6
	SVM	85.43%	0.076	21.46%	7

The classification accuracy is a discretized result based on engineering standard thresholds and is highly sensitive to samples near the boundaries. Even with low numerical errors, samples near thresholds may lead to reduced classification accuracy. Therefore, based on the practical needs of engineering calculations and the sensitivity of parameters to continuous variables, this study prioritizes error metrics as the primary criterion, with classification accuracy serving as an auxiliary constraint, ensuring the model possesses both numerical precision and engineering usability.

As shown in Table 11, the performance ranking of the models is consistent across the three counties. Non-linear models (MLP, RF, XGBoost) generally outperform the linear regression model, indicating a significant non-linear coupling relationship between loess compressibility and multi-indices. Under the principle of “error metrics priority, classification accuracy auxiliary,” the MLP model achieved the lowest mean error and relative error in Huocheng, Nilka, and Xinyuan, demonstrating the best comprehensive deviation control capability, thus being identified as the optimal model; the RF model followed, showing good robustness.

From a mechanistic perspective, MLP relies on multi-layer non-linear mapping to represent complex input-output relationships in high-dimensional feature space. RF reduces variance and overfitting risk via Bootstrap integration and random feature subsets, making it more robust to noise and outliers, which is advantageous for engineering classification stability. CART, as a single-tree model, is interpretable but limited in capturing complex non-linearity and generalization. XGBoost offers high precision via gradient boosting but is sensitive to hyperparameters. The linear regression model is constrained by linearity assumptions and potential multicollinearity. RBF and SVM are sensitive to kernel or structural parameters, leading to performance fluctuations with varying sample sizes and feature scales.

In summary, under the sample scope and indicator system of this study, MLP performs as the optimal model in most counties, followed by RF. The two can complement each other in different application scenarios: MLP is preferred for continuous value prediction and error control, while RF is preferred for robustness in engineering classification.

## 5. Conclusion

Based on the physical, hydraulic, and mechanical parameters of collapsible loess in the Ili region, this study selected Huocheng, Nilka, and Xinyuan counties as typical study areas. By employing statistical methods, Multiple Linear Regression (MLR), and machine learning methods, correlations were analyzed, and prediction models were established and verified. The main conclusions are as follows:

(1) The correlation between loess compressibility and soil properties in the Ili region was analyzed. The analysis results indicate that the key factors influencing loess compressibility are physical-mechanical indicators. The compression coefficient a is significantly positively correlated with pore structure indices (void ratio *e*) and negatively correlated with dry density *ρ*_*d*_ and modulus of compressibility *E*_*s*_. Considering correlation strength, significance tests, and engineering accessibility, *E*_*s*_, *ρ*_*d*_, and *e* were selected as core input indicators.
(2) Prediction models for loess compressibility in the Ili region were established. Using multiple regression and machine learning theories, prediction models (including MLR, MLP, RF, RBF, SVM, CART, and XGBoost) were constructed for Huocheng, Nilka, and Xinyuan counties using *E*_*s*_, *ρ*_*d*_, and *e* as input indicators. These models can successfully predict the compression coefficient a and its engineering classification within the scope of this study, providing methodological support for the rapid estimation of compressibility parameter.
(3) The prediction evaluation indicators and model performance for loess compressibility in the study area were analyzed. Under the evaluation principle of “error metrics priority, classification accuracy auxiliary,” the MLP model achieved the best overall error metrics across all three counties, demonstrating the best comprehensive deviation control capability. The RF model showed superior stability and robustness, ranking second. Therefore, under the sample scope and county calibration conditions of this study, the MLP model is recommended as the optimal model, with RF as an engineering alternative. Future applications should consider local calibration to reduce extrapolation uncertainty.
